# Adulteration Identification of Angelica Sinensis Radix Based on Molecular Matrix Characteristics

**DOI:** 10.3390/foods14173005

**Published:** 2025-08-27

**Authors:** Yu Zhang, Xiaohan Guo, Lizhi Wan, Jiating Zhang, Wenguang Jing, Minghua Li, Xianlong Cheng, Feng Wei

**Affiliations:** 1 Institute for Control of Traditional Chinese Medicine and Ethnic Medicine, National Institutes for Food and Drug Control, Beijing 102629, China; zhangyu55505@163.com (Y.Z.); guoxiaohan@nifdc.org.cn (X.G.); wanlizhi@nifdc.org.cn (L.W.); 18341441178@163.com (J.Z.); jingwenguang@nifdc.org.cn (W.J.); liminghua@nifdc.org.cn (M.L.); 2 Faculty of Functional Food and Wine, Shenyang Pharmaceutical University, Shenyang 110016, China; 3 State Key Laboratory of Drug Regulatory Science, National Institutes for Food and Drug Control, Beijing 102629, China

**Keywords:** adulteration identification, angelica sinensis radix, digitally identify, UHPLC-QTOF-MS

## Abstract

Angelica sinensis radix (AS), the dried root of *Angelica sinensis* (Oliv.) *Diels*, is widely used in Chinese medicine and food products. However, after conducting market research, at least a quarter of AS on the market is commonly adulterated by *Levisticum officinale* W. D. J. Koch (LO), *Angelica acutiloba* (Sieb. et Zucc.) Kitagawa (AA), and *Angelica gigas* Nakai (AG), to varying degrees, which significantly affects its clinical efficacy and food safety. Therefore, there is a pressing need to establish safe and reliable methods for identifying illicit adulteration. In this study, the mass spectrometry (MS) information of AS, LO, AA, and AG was collected and converted into the data matrix for [*t_R_-m/z*-I]. The *top-n* proprietary ions of AS, AG, AA, and LO were output as their molecular “matrix characteristics”. Test samples were also analyzed, transformed into data matrices, and their own matrix characteristics were matched sequentially. For matching credibility (MC) results, a significant difference was found between the MC of the four herbs compared to their own matrix characteristics, as well as between the MC of the four herbs compared with their non-self matrix characteristics. Research results showed that based on matrix characteristics, AS and its adulterations can be identified with a matching credibility (MC) ≥ 78.0%; 3% adulterations can also be identified, and two market-blind samples were identified as exhibiting adulterations. In addition, chemometrics analysis demonstrated that adulteration identification based on matrix characteristics is reasonable and reliable. The matrix characteristics of AS and its adulterants contribute to adulteration analysis. The identification method, based on matrix characteristics, is safe and reliable which is conducive to AS’s quality control and market supervision.

## 1. Introduction

Angelica sinensis radix (AS) is the dried root of *Angelica sinensis* (Oliv.) Diels., exhibiting both food and medicinal properties. On the one hand, it can be used as a flavoring agent and eaten after washing and soaking to soften; on the other hand, besides being used as a condiment, Angelica sinensis radix (AS) can also be processed into various foods, such as soups, stews, and steamed dumplings. As a traditional “warming and tonifying” Chinese medicine, AS has the effect of regulating menstruation and tonifying blood [1,2,3]. However, some adulterants of AS are on the market. LO is the dried root of *Levisticum officinale* W. D. J. Koch of Umbelliferae *Angelica*. AA is the dried root of *A. acutiloba Kitagava* and *A. acutiloba Kitagava* var—Sugiyama *Hikino* of Umbelliferae. In addition, AG is the dried root of *Angelica gigas* Nakai. In the Chinese pharmacopoeia, only AS can be used as a medicinal herb. However, due to their similar macroscopic and microscopic characteristics, LO, AA, and AG are typical umbelliferae adulterants of AS. In the Chinese medicine and food market, LO, AA, and AG are often regarded as AS or mixed into AS, affecting not only AS’s taste and aroma but also the market supervision, quality control, and clinical application of AS [4,5,6]. Therefore, it is crucial to compare and distinguish the four herbs from many aspects and establish a method of adulteration identification.

Fu Hong identified AS, AA, and LO based on microscopic trait characterization and thin-layer chromatography (TLC) [7]. Wang YD et al. used HPLC fingerprinting combined with chemometric analysis to observe the chemical compositional differences between AS and LO. The results showed that the critical components for distinguishing AS from LO were Falcarindiol, Tryptophan, E-ibutilide, and Chlorogenic acid [8]. Zhou SS et al. integrated metabolomics and glycomics to evaluate AS and AA, which facilitated the identification of AA and AS [5]. Ahn SJ et al. established the discrimination model using LC-QTOF/MS and PLS-DA, and the results showed that the PLS-DA model can effectively distinguish AS, AG, and AA [9]. Regarding molecular identification, Minami, M. et al. used DNA sequences to identify AA and AS [4]. The above research is beneficial to the identification and analysis of AS and its adulterants (LO, AA, AG).

However, microscopic identification requires professional skills and can be subjective. At the same time, the main methods of chemical identification are HPLC and HPLC-MS, which focus on a few known chemical markers (quality control markers), both of which can distinguish different varieties of AS; however, they fail to make reasonably practical use of information about unknown chemical compositions [10]. More importantly, microscopic trait characterization and the main methods of chemical identification are more suitable for comparing genuine products with counterfeits but not sufficient for detecting adulterated samples. Although DNA barcodes can identify adulterated samples, DNA is easily degraded in the sample preparation process, and the sample is preferably fresh, which limits the application of DNA barcode technology [11].

Based on the current identification analysis, we have summarized the advantages and disadvantages of various identification methods. Table 1 shows the advantages and disadvantages of various identification methods.

Given the above reflections, as well as the enrichment of the identification method of AS and its adulterants [12], in this study, we constructed “matrix characteristics” of AS, AG, AA, and LO (belonging to the field of mass spectrometry analysis) from the perspective of the mass spectrometry of chemical compositions to identify AS and its adulterants. In addition, the results of the matrix characteristics-based identification method were validated through chemometrics.

## 2. Materials and Methods

### 2.1. Herbal Materials

Samples of 10 AS, as well as 10 LO, were sourced from different origins in China; 6 AG and 6 AA were sourced from different origins, including Japan, China, and Korea, and Professor Feng Wei identified all the above herbs, ensuring that they met the variety requirements. A total of 21 positive adulterated samples, including 0% LO, 3% LO, 5% LO, 10% LO, 20% LO, 50% LO, 100% LO, 0% AG, 3% AG, 5% AG, 10% AG, 20% AG, 50% AG, 100% AG, 0% AA, 3% AA, 5% AA, 10% AA, 20% AA, 50% AA, and 100% AA [6,13] were included in the study. In addition, nine AS herbal materials were sourced from different herbal companies. Table S1 shows the herbal information.

### 2.2. Experimental Consumables

Acetonitrile (Lot: Y5BA1H) was purchased from Thermo Fisher Technology Co., Ltd. (Waltham, MA, USA) Methanol (Lot: EI606-US), and Honeywell Trading Co., Ltd. (Charlotte, NC, USA) supplied the formic acid (Lot: 50153). Guangzhou Watsons Food and Beverage Co., Ltd. (Guangzhou, China) provided the ultrapure water (Lot: GB19298). The 2 mL disposable syringe (Lot: 240115) was purchased from Shanghai Anpu Experimental Technology Co., Ltd. (Shanghai, China). Merck Millipore Co., Ltd. (Burlington, MA, USA) provided the 0.22 μm organic filter membranes (Lot: 0392122).

### 2.3. Mass Spectrometry Conditions

A Waters Xevo G2-XS QTOF mass spectrometer with an electrospray ionization (ESI) ion source; positive and negative ion mode; data acquisition mode of MS^E^; scan time set to 0.1 s; and mass-to-charge ratio (*m/z*) of 100~1500 Da was used in this study. To effectively separate the components and obtain stable ion signals, this experiment explored suitable mass spectrometry conditions, i.e., capillary voltage (default parameters): 3.0 kV (ESI+), 2.5 kV (ESI-); cone (default parameter): 40 eV; source bias (default parameter): 80 eV; desolventization temperature (default parameter): 450 °C; desolventizing gas flow rate (default parameter): 600 L/h; collision: 10~40 eV (more chemical composition information); ion source temperature (default parameters): 120 °C [6]. Before sample analysis, sodium formate and leucine enkephalin (LE, 300 ng·mL^−1^) were utilized for mass axis and LockSpray correction; During UHPLC-QTOF-MS^E^ analysis, LE was utilized for real-time mass correction.

### 2.4. Chromatographic Conditions

A Waters ACQUITY UPLC System and a Waters UHPLC BEH-C18 chromatographic column (2.1 mm × 100 mm, 1.7 μm) were used in this study, with column temperature, 35 °C; sample tray temperature, 15 °C; injection volume, 2 μL; mobile phase, acetonitrile (C), 0.1% formic acid aqueous solution (D); flow rate, 0.3 mL/min; and gradient elution, 0 min, 5% C; 23.0 min, 95% C; 26.0 min, 95% C; 26.1 min, 5% C; 30.0 min, 5% C [13].

### 2.5. Sample Pretreatment

All the herbs were pulverized and filtered through a No. 3 sieve. All batches of AS powder were mixed in equal proportions as those used for the AS reference samples. At the same time, LO, AA, and AG reference samples were prepared using the same method. Further, different proportions of LO, AA, and AG were added to AS (0%, 3%, 5%, 10%, 20%, 50%, 100%) to obtain mixed positive samples, respectively [6,13]. Finally, each sample was accurately weighed at 1.00 g, placed in a 50 mL conical flask with a stopper, and 50.00 mL of methanol was accurately added to the mixture, which was then ultrasonicated for 30 min under 450 W and 40 kHz conditions. Then, the test samples were obtained by filtering through a 0.22 μm membrane [13].

### 2.6. Digital Identification Preparation

Each test sample’s and blank’s MS was converted into a [*t_R_-m/z*-I] data matrix. The form is as follows [13]:

(1) $$\mathrm{Blank}\;\mathrm{or}\;\mathrm{TCM}\;(\mathrm{AS}\;\mathrm{or}\;\mathrm{AA}\;\mathrm{or}\;\mathrm{LO}\;\mathrm{or}\;\mathrm{AG})\;=\;\left[\begin{array}{ccc} t_{R} & m/z & I\\ ••• & ••• & •••\\ t_{n} & m_{n} & i_{n} \end{array}\right]$$

**Figure 1:**
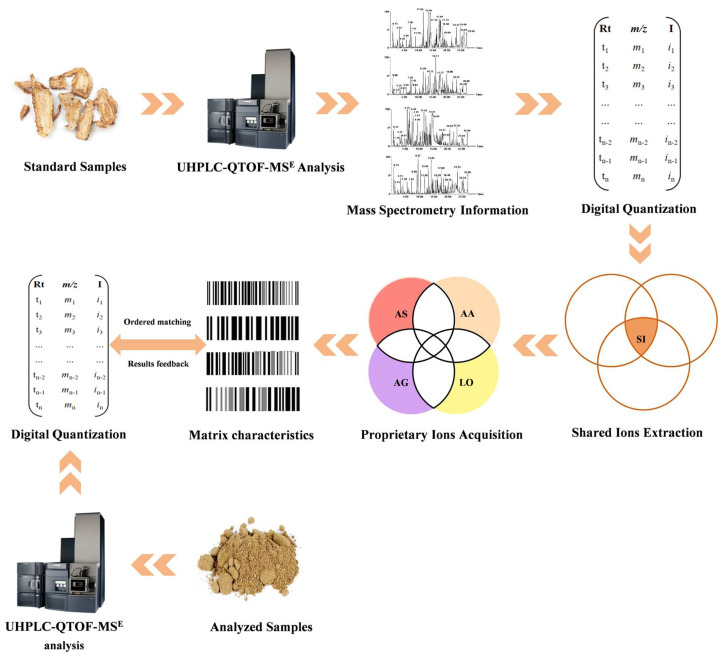
The diagram of the identification process.

As shown in Figure 1, the identification flow is as follows [6,13]: Firstly, AS, AG, AA, and LO samples were analyzed by mass spectrometry to acquire their MS information. Secondly, the MS information was converted into the [tR-m/z-I] data matrix. Thirdly, the consensus ions were extracted from multiple batches of AS, AG, AA, and LO samples for their “ion characterization”, respectively. Further, the proprietary ions of AS, AG, AA, and LO were acquired by removing the consensus ions. Finally, the top-n ions were selected as the matrix characteristics of AS, AG, AA, and LO, sorted by ion intensity. The matrix characteristics of AS, AG, AA, and LO were used for matching unknown samples to provide feedback on matching credibility (MC).

In the identification process, the specific algorithm flow is as follows [6,13]. (1) Background ions deduction: If the *t_R_* and *m/z* of ions in the samples are similar to those in methanol, Δ*t_R_* ≤ 0.20 min, and Δ*m/z* ≤ 0.01 Da, these ions are considered as interfering ions and are removed from the ion matrix. (2) Acquisition of consensus ions: If the *t_R_* and *m/z* of ions in multiple batches of the same herb samples satisfy Δ*t_R_* ≤ 0.20 min and Δm/z ≤ 0.01 Da, these ions are considered as consensus ions. (3) Acquisition of proprietary ions: The AS data of the consensus ions is compared with the raw MS data of AA, AG, and LO to obtain AS’s proprietary ions. The specific AG, AA, and LO ions can be obtained through the same method. The non-specific ions satisfy Δ*t_R_* ≤ 0.20 min and Δ*m/z* ≤ 0.01 Da during the screening process. (4) Acquisition of matrix characteristics: In the proprietary ions obtained, the top 100 ions are taken as the matrix characteristics of AS, AA, LO, and AG, respectively, in the order of ion abundance. (5) Identification analysis: The matrix characteristics of AS, AG, AA, and LO are employed to match the test samples, using MC to realize identification. If the ion satisfies Δ*t_R_* ≤ 0.20 min and Δ*m/z* ≤ 0.01 Da, it is considered a successful ion match.

(2) $$\mathrm{MC}=\frac{\begin{array}{c} number\;of\;matched\;ions \end{array}}{\begin{array}{c} number\;of\;ions\;in\;matrix\;characteristics \end{array}}\times 100$$

## 3. Results

### 3.1. Results of UHPLC-QTOF-MS^E^ Analysis

**Figure 2:**
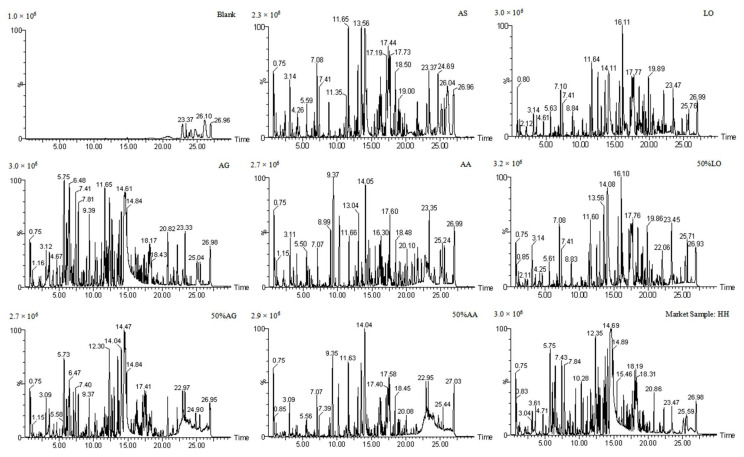
The base peak chromatograms of AS, AG, AA, LO (AS: Angelica sinensis radix, AG: *Angelica gigas* Nakai, AA: *Angelica acutiloba* (Sieb. et Zucc.) Kitagawa, LO: *levisticum officinale* W. D. J. Koch), positive samples, and market herbs.

The base peak chromatograms after the same treatment and analysis are shown in Figure 2. The blank does not interfere with the test samples. Moreover, the ion chromatograms of AS, AA, LO, and AG are not similar under the same conditions. Therefore, we can identify a single herb by comparing the ion chromatograms. However, ion chromatograms comparison does not apply to the identification of adulteration samples. Therefore, digital identification and extraction of matrix characteristics are necessary for adulteration identification.

On the other hand, we examined and optimized the analytical conditions. First, the positive and negative ion modes were compared, and the results showed that more mass spectrometry information reflecting the chemical composition was available in the positive mode, and the ion intensity was higher. Some chemicals are lost, which can only be detected in negative ion mode. However, this does not affect the final appraisal result. Furthermore, the removal of the negative ion mode can prevent the interference of low-quality data (low ion intensity in ESI−), effectively save time, and increase throughput. It is perfectly fine to consider both positive and negative ions at the same time, but in actual application analysis, each test sample would require data collection in both positive and negative ion modes, which would be time-consuming and impractical. Adulteration is effectively analyzed based on the ordered molecular combinations in the positive ion mode alone. Secondly, methanol provided the best extraction compared to dichloromethane, 50% methanol–water, and water.

### 3.2. Results of Identification of AS, AA, AG, and LO

The [*t_R_-m/z*-I] results of the matrix characteristics are shown in Tables S2–S5. The AS, AA, and LO test samples were also analyzed and transformed into data matrices, and their own matrix characteristics were matched sequentially. The higher the MC, the greater the similarity between the test sample and the medicinal material, and the greater the possibility that the sample is the medicinal material. Table 2 shows the results of MC. The matching credibility (MC) of AS, AA, LO, and AG matched with their own matrix characteristics was higher than 77%. At the same time, the matching credibility (MC) of AS, AA, LO, and AG matched with their non-self matrix characteristics was not higher than 5%. Further, non-parametric tests showed a significant difference between the MC of the four herbs compared to their own matrix characteristics and the MC of the four herbs compared with their non-self matrix characteristics (*p* < 0.003).

In identifying the four herbs, we also examined the effects of ion number and data bias. The matching results in different numbers of ions are summarized in Table 3, with the top 50, 100, 150, and 200 ions output as matrix characteristics. As seen from the matching results in Table 3, more output ions do not indicate a better match. After comprehensive consideration, the matrix characteristics with top 100 ions ensure a high recognition of adulterants without affecting the identification of AS. In addition, sample data were collected by different personnel at different time rather than at the same time, fully considering the effects of instrument status and operation by different personnel. However, it was found that the *t_R_* and *m/z* did deviate, but the retention time did not deviate by more than 0.2 min, and the *m*/*z* did not deviate by more than 0.01 Da. Therefore, we finalized the *t_R_* and *m*/*z* as Δ*t_R_* ≤ 0.20 min and Δ*m/z* ≤ 0.01 Da, respectively.

### 3.3. Results of Adulterant Analysis

#### 3.3.1. Results of Mixed Sample Identification

**Figure 3:**
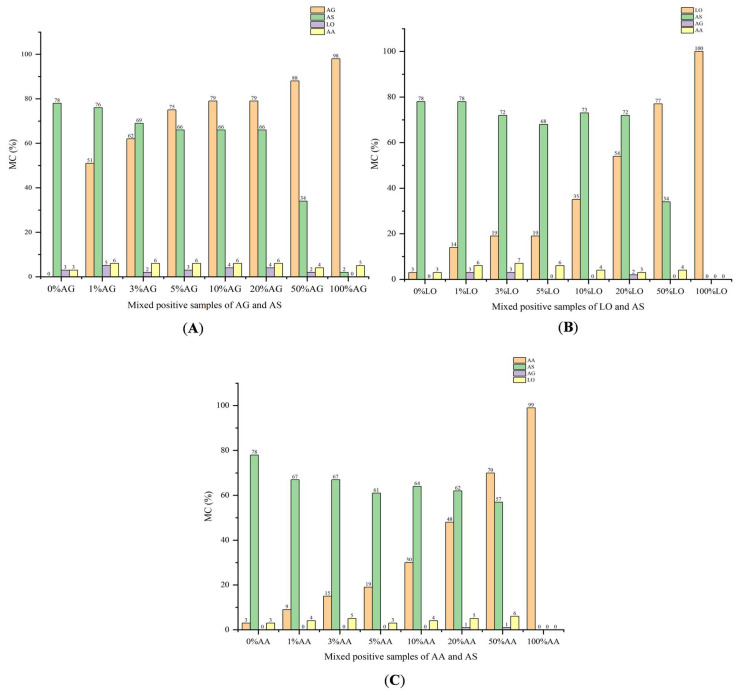
The matching results of mixed positive samples. (**A**): The results of mixed positive samples of *Angelica gigas* Nakai (AG) and Angelica sinensis radix (AS); (**B**): the results of mixed positive samples of *levisticum officinale* W. D. J. Koch (LO) and Angelica sinensis radix (AS); (**C**): the results of mixed positive samples of *Angelica acutiloba* (Sieb. et Zucc.) Kitagawa (AA) and Angelica sinensis radix (AS).

Figure 3 shows the matching results. Figure 3A shows that the MC of mixed samples compared to the matrix characteristics of AA and LO were not higher than 6% for mixed AG and AS adulterated samples. Meanwhile, the MC of mixed samples compared to the matrix characteristics of AG and AS were not less than 34%. This showed at least a 5-fold difference, which suggests that matrix characteristics of AS, AA, AG, and LO express a certain degree of specificity. For the identification of AG and AS, as the proportion of AG in the mixed samples increased, the MC of AG showed an upward trend when matched with AG’s matrix characteristics. The MC of 1% AG is the most minor, expressing at least 51%, and the MC of 100% AG is 98%. At the same time, the MC of 0% AG compared to its own matrix characteristics is 0%. On the other hand, from 0% AG to 50% AG, the MC was all higher than 34% when matched with the AS’s matrix characteristics. Meanwhile, the MC of 100% AG compared to AS’s matrix characteristics is only 2%. Therefore, it can be seen that MC can effectively reflect the degree of adulterated AG in AS without affecting AS’s identification.

Figure 3B shows the identification results of mixed samples of LO and AS. For mixed samples of LO and AS, the MC of mixed samples compared to the matrix characteristics of LO and AS were also not less than 34%, and the MC of mixed samples compared to AG and AA’s matrix characteristics was not higher than 7%. This showed at least a 4-fold difference, suggesting that based on the matrix characteristics of LO and AS, the identification of LO in AS can be realized. In addition, as the proportion of LO in the mixed samples increased, MC was also significantly more prominent. The MC of 1% LO and 100% LO was 14% and 100%, respectively. However, the MC of 0% LO compared to LO’s matrix characteristics was only 3%. As shown in Figure 3C, from 1% AA to 100% AA, the MC of the samples compared to the matrix characteristics of AA was all not less than 9% for mixed positive samples of AA and AS. At the same time, the MC of 0% AA was only 3%. On the other hand, from 0% AA to 50% AA, the MC of samples compared to the AS’s matrix characteristics was not less than 57%. However, the MC of 100% AA compared to AS’s matrix characteristics was only 0%, demonstrating that AA adulteration can be identified based on AA and AS’s matrix characteristics, without affecting AA’s identification. The above study shows that the matrix characteristics of AS, AA, AG, and LO show a certain degree of specificity to realize adulterant identification.

Based on the identification results of the positive adulterated samples, considering that a total of 3% impurities is allowed in herbs, the MC value measured when 3% AG, LO, and AA were doped into the AS sample was selected as the detected threshold. We initially set the detection limit for MC of AG to 62%. Similarly, for LO and AA, we initially set the detection limit of MC as 19% and 15%, respectively. In identifying unknown samples, if the MC is much greater than the set threshold, we can initially conclude that the sample is adulterated with AG, LO, or AA. If the MC is slightly higher than the detection threshold (threshold + 5%), the corresponding samples are temporarily regarded as suspicious samples.

#### 3.3.2. Adulterant Identification of Market Blind Samples

**Figure 4:**
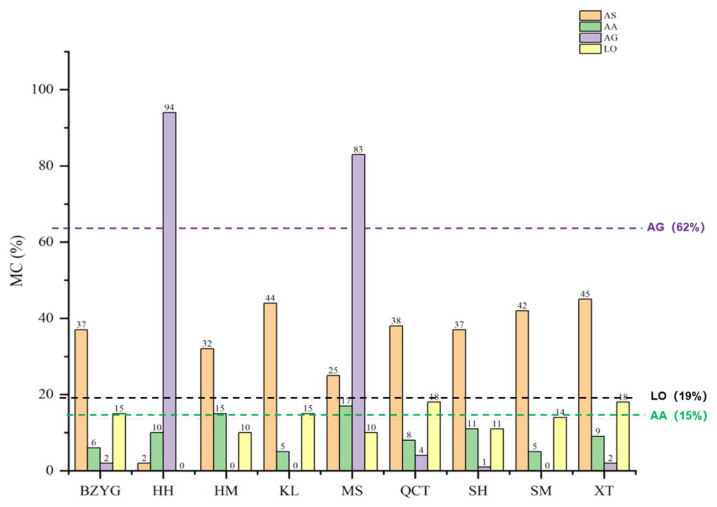
The matching results of market blind samples (market blind sample lot numbers: BZYG, HH, HM, KL, MS, QCT, SH, SM, XT).

Based on the matrix characteristics of AS, AA, AG, and LO, we analyzed nine batches of marketed AS samples for adulteration. Figure 4 shows that the MC of HH and MS materials compared to AG’s matrix characteristics was 94% and 83%, respectively, much greater than AG’s MC detection limit (62%). Therefore, we can conclude that the AS market samples from HH and MS were adulterated with AG based on AG’s matrix characteristics. As for the identification situation of LO, the MC of all market herbal materials is below the MC detection limit of LO. It showed no adulteration of LO in the nine batches of AS market samples. As for the AA identification, the MC of HM and MS samples was 15% and 17%, respectively, which can be regarded as suspicious results.

### 3.4. Chemometric Analysis of Adulterant Identification

**Figure 5:**
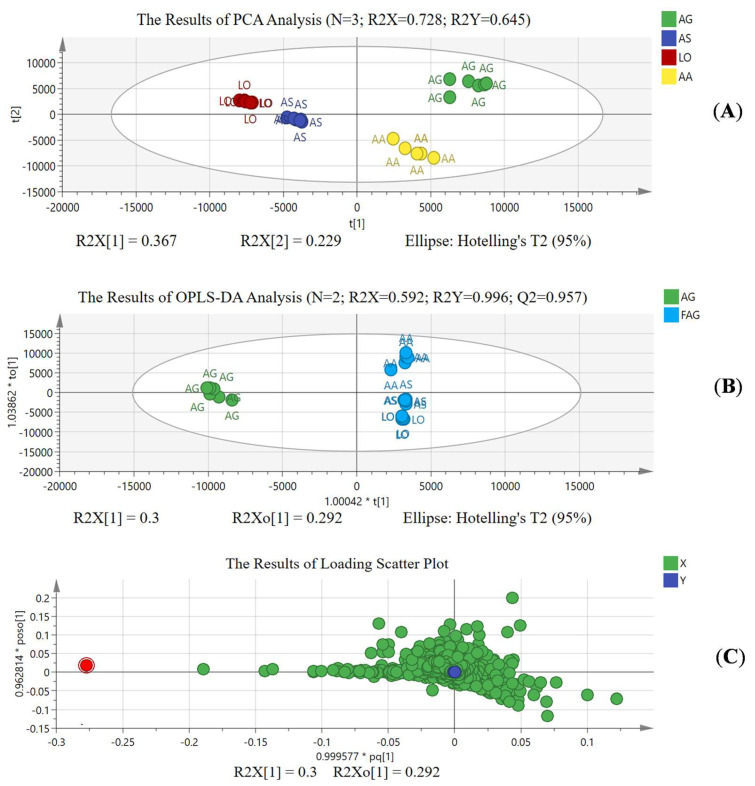
The results of chemometric analysis of AG and non−AG. (**A**): The results of PCA analysis; (**B**): the results of OPLS−DA analysis; (**C**): the results of loading scatter plot.

Identification based on matrix characteristics alone is not sufficiently convincing, especially regarding suspicious samples, as the market sample is a blind sample. Therefore, chemometric analyses were carried out to explore the differential components for adulteration identification, validating the results based on matrix characteristics. A total of 23 batches of AS, AA, AG, and LO standard samples were analyzed using PCA, OPLS-DA, and S-plot [14,15,16]. Figure 5 shows the results of the chemometric analysis. As shown in Figure 5A, via PCA analysis, the AS, AA, AG, and LO could distinguish each other with R^2^X = 0.728 and R^2^Y = 0.645, based on the three principal components. The results of PCA analysis showed that AS, AA, AG, and LO have differentiated chemical compositions. Further, we divided the sample data into AG and non-AG groups for OPLS-DA analysis to explore the differential components of AG. Figure 5B shows that AG can be distinguished from AA, AS, and LO through two principal components, R^2^X = 0.592, R^2^Y = 0.996, and Q^2^ = 0.957 in OPLS-DA analysis. The OPLS-DA model exhibits practical significance [15,17]. As shown in Figure 5C, the loading scatter plot shows the extent to which the chemical compositions (data points) contribute to the distinction between AG and non-AG. Generally speaking, data points far from the origin are essential to distinguish between AG and non-AG. In Figure 5C, the red point (14.75 min_329.1395 *m/z* [M + H]^+^) far from the origin represents the differential chemical component highly abundant in the AG but at baseline the AA, AS, and LO results.

**Figure 6:**
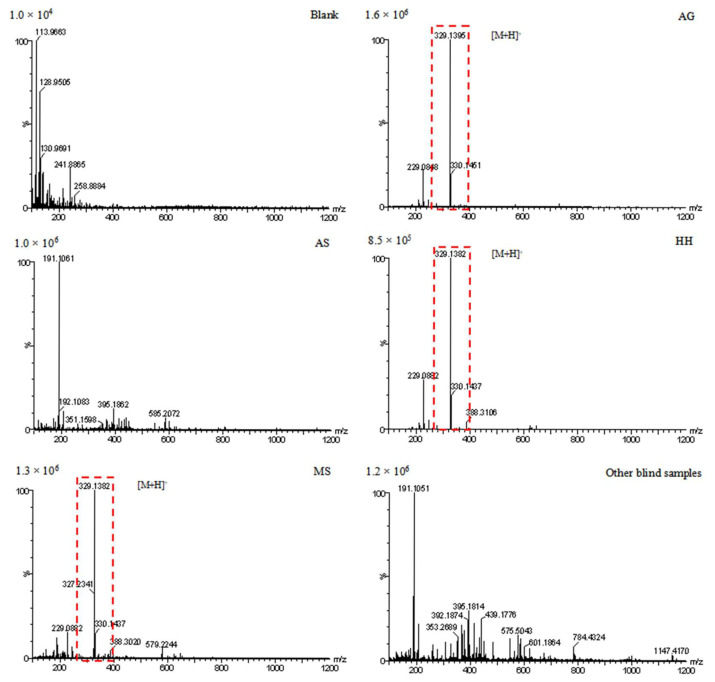
The extraction results of 14.75 min_329.1395 *m/z* in different samples.

Based on the chemometric analysis of AG and non-AG, the chemical component (14.75 min_329.1395 *m/z*) is taken as the chemical marker of AG to extract the mass spectrometry of the blank, AS, AG standard samples, and AS market blind samples. Figure 6 shows the extraction results. The characteristic peak can be extracted from the AG standard samples and the HH and MS blind samples. Meanwhile, it cannot be extracted from the blank, AS standard samples, or other market-blind samples. The above analyses indicate that the HH and MS blind samples were adulterated with AG, consistent with the identification based on the matrix characteristics of AS and AG.

In addition, as shown in Figures S1 and S2, using the same chemometric analysis, we obtained the differential chemical composition (10.31 min_247.0621 *m/z* [M + H]^+^) for AA and the differential chemical composition (16.10 min_727.5131 *m/z* [M + H]^+^) for LO, respectively. The characteristic ion (10.31 min_247.0616 *m/z* [M + H]^+^) can be found in the AA samples, and the ionic abundance is greater than 4.5 × 10^5^. However, its ionic abundance is at baseline levels in the blank, AS samples, and all the market blind samples, including the HM and MS suspicious samples. No AA was adulterated in the nine batches of blind market samples. The extraction results for 10.31 min_247.0621 *m*/*z* are shown in Figure S3. As for the differential ion (16.10 min_727.5131 *m/z* [M + H]^+^) for LO, it can be found in AS and LO, but the ionic abundance displays a significant difference. As shown in Figure S4, its ionic abundance in the LO samples is higher than 1.6 × 10^7^, but its ionic abundance in the AS samples is less than 2.5 × 10^5^. At the same time, the ionic abundance for 16.10 min_727.5095 *m/z* in the 3% LO mixed sample is 1.8 × 10^6^. Moreover, the ionic abundance increased as the proportion of LO in the mixed sample increased. For the 50% LO mixed sample, its ionic abundance is 5.9 × 10^6^. Therefore, 16.10 min_727.5131 *m/z*, although not a proprietary ion, can still help determine whether or not adulteration is present in a blind sample through the comparison of ion abundance. The ionic abundance of 16.10 min_727.5095 *m/z* in the AS blind samples was not higher than 9.0 × 10^5^. Therefore, it is reasonable to assume that the AS blind market samples were not adulterated with LO.

In summary, based on the chemometric analysis, we can determine that the AS blind samples of HH and MS were indeed adulterated with AG. It also proves that adulteration identification based on the matrix characteristics of AA, AS, LO, and AG is reasonable and reliable.

## 4. Discussion

Compared with using a few chemical components as quality control indexes, introducing the concept of a mathematical matrix into the adulterant identification of TCM and constructing the quantized matrix characteristics of chemical components exhibits a more robust proprietary nature. It enables non-targeted adulteration identification through the matrix characteristics of AA, AS, AG, and LO. However, obtaining the representative matrix characteristics for AA, AS, AG, and LO is crucial, as is the consideration of the diversity and representativeness of the samples. Therefore, the samples used in this study were standardized samples from different origins, personally harvested by laboratory staff and confirmed by researcher Wei Feng as meeting the species requirements. On the other hand, chemical constituents are essential molecules in herbal medicines and are the basis of their efficacy and flavor. Therefore, we constructed matrix characteristics using quantized data of chemical compositions. Meanwhile, we obtained a quantized characterization of chemical components based on high-resolution mass spectrometry because it can provide massive amounts of data in a limited detection time [18,19].

Compared to method involving the identification for the DNA barcode [11,20], this study method requires fewer analytical processes, with no need to identify chemical compositions, which can significantly improve the efficiency of identifying AS adulteration. Moreover, due to the potential degradation of DNA, DNA barcoding is often performed using fresh products [11]. In contrast, chemical-based matrix characteristics are not affected by chemical degradation. Because matrix characteristics focus on the shared chemical composition of different origins and batches of the same herb, only chemical components detected in multiple batches of the same herb from various origins are targeted. On the other hand, this study still displays some shortcomings: although the samples cover different origins and harvest periods, the number of samples is small. More samples need to be collected for exploration. In the chemometric validation of adulterant identification, we were not able to precisely determine the compounds of the three differential chemical components, but this did not prevent us from conducting the validation. Of course, the identification of the three compounds will render the identification work more accurate and persuasive, along with making its application to market management more convenient. This is also our future research direction.

## 5. Conclusions

Based on UHPLC-QTOF-MS^E^ analysis and quantized characterization, the matrix characteristics of AS, AA, AG, and LO were constructed to conduct adulterant identification. Adulterations as small as 3% can also be identified, and two market-blind samples were identified as adulterations. Moreover, chemometric analysis has proven that adulteration identification based on matrix characteristics is reasonable and reliable. The specific processes are as follows: firstly, digital matching is carried out based on digital identity, and a preliminary judgment is made according to the MC feedback value; then an accurate judgment is made based on chemometrics analysis. Quality supervision in herbal medicine is a key measure to ensure the safety of public medications. This research provides scientific and technological means for identifying the illegal addition of some substances to foods and drugs; maintaining a safe production environment; ensuring food and drug safety; and guaranteeing the effectiveness of supervision, which is conducive to the quality control of food and drugs. It also promotes the digitalization process of supervision technology.

## Figures and Tables

**Table 1:** The advantages and disadvantages of various identification methods.

Identification Methods	Advantages	Disadvantages
Trait characterization	Simple, fast, and low cost; good intuitiveness; practical applications.	Highly subjective; difficulty distinguishing between closely related species; high professional requirements.
Microscopic characterization	Objective and stable; detects subtle differences; small sample size.	High professional requirements; low efficiency; limited applicability.
TLC/HPLC	Standardizability; reproducibility; widely applicable; provides complex and quantitative analysis.	Handles tedious tasks; limited information requirements; single or few ingredients; poor specificity.
LC-MS	Multi-component; trace detection; highly specialized and sensitive; widely applicable; high accuracy.	Handles tedious tasks; high cost; technically challenging; database-dependent; standardization gap; unknown chemical composition not fully utilized.
DNA	High accuracy; highly proprietary; highly objective and reproducible; high sensitivity; small sample size.	DNA degradation and damage; technically complex and relatively expensive; database-dependent.

**Table 2:** The results of matching credibility results of AS, AA, AG, and LO test samples.

Name	Batch	MC (AS)	MC (AG)	MC (AA)	MC (LO)
AS	AS10	78%	0%	3%	3%
AG	AG06	2%	98%	5%	0%
AA	AA06	0%	0%	90%	0%
LO	LO10	4%	0%	1%	99%

**Table 3:** The matching results based on matrix characteristics with different numbers of ions.

Name	Batch	50 Ions	100 Ions	150 Ions	200 Ions
AS	AS10	72%	78%	79%	79%
AG	AG06	98%	99%	95%	95%
AA	AA06	94%	98%	97%	97%
LO	LO10	88%	90%	91%	92%

## Data Availability

Data will be made available on request.

## Supplementary Materials

The following supporting information can be downloaded at: https://www.mdpi.com/article/10.3390/foods14173005/s1, Table S1: The detailed information of herbal materials; Table S2: The ion information of matrix characteristics of *Angelica gigas* (AG); Table S3: The ion information of matrix characteristics of *Angelica Acutiloba* (AA); Table S4: The ion information of matrix characteristics of *Levisticum Officinale* (LO); Table S5: The ion information of matrix characteristics of *Angelica Sinensis* (AS); Figure S1: The results of chemometric analysis of AA and non-AA; Figure S2: The results of chemometric analysis of LO and non-LO; Figure S3: The extraction results of 10.31 min_247.0621 *m/z* in different samples; Figure S4: The extraction results of 16.10 min_727.5131 *m/z* in different samples.

## Abbreviations

The following abbreviations are used in this manuscript:

AA	*Angelica acutiloba* (Sieb. et Zucc.) Kitagawa
AG	*Angelica gigas* Nakai
AS	Angelica sinensis radix
DM	digital matrix
LO	*Levisticum officinale* W. D. J. Koch
MC	matching credibility
MS	mass spectrometry
*m*/*z*	mass-to-charge ratio
OPLS-DA	orthogonal partial least squares discriminant analysis
PCA	principal component analysis
TCM	traditional Chinese medicine
