# Supplementary material for: Adulteration Identification of Angelica Sinensis Radix Based on Molecular Matrix Characteristics
Source: Foods. 2025 Aug 27;14(17):3005. doi: 10.3390/foods14173005 (PMC12428496; doi:10.3390/foods14173005)
Supplement: Supplementary file 1 [file foods-14-03005-s001.zip › foods-3799152-supplementary.pdf]

Adulteration Identification of Angelica Sinensis by TCM database of LC-MS Matrix Characteristics

Yu Zhang[1,2], Xiaohan Guo[1,3†], Lizhi Wan[1,3†], Jiating Zhang[1,3], Wenguang Jing[1,3], Minghua Li[1,3], Xianlong Cheng[1,3*], Feng Wei[1,3*]

†These authors contributed equally to this work and should be listed as the co-first authors

[1]Institute for Control of Traditional Chinese Medicine and Ethnic Medicine, National Institutes for Food and Drug Control, Beijing, P. R. China 102629

[2]Faculty of Functional Food and Wine, Shenyang Pharmaceutical University, Shenyang, 110016 China

[3]State Key Laboratory of Drug Regulatory Science, National Institutes for Food and Drug Control, Beijing 102629, China

Table S1 The detailed information of herbal materials

Herbal materials	Batch	Sample Collection/Preparation Unit	Use	Place of origin
Angelica Sinensis	AS01	National Institutes for Food and Drug Control	"digital identity"; mixed sample	Gansu, China
Angelica Sinensis	AS02	National Institutes for Food and Drug Control	"digital identity"; mixed sample	Sichuan, China
Angelica Sinensis	AS03	National Institutes for Food and Drug Control	"digital identity"; mixed sample	Yunnan, China
Angelica Sinensis	AS04	National Institutes for Food and Drug Control	"digital identity"; mixed sample	Shanxi, China
Angelica Sinensis	AS05	National Institutes for Food and Drug Control	"digital identity"; mixed sample	Hubei, China
Angelica Sinensis	AS06	National Institutes for Food and Drug Control	"digital identity"; mixed sample	Shan'Xi, China
Angelica Sinensis	AS07	National Institutes for Food and Drug Control	"digital identity"; mixed sample	Yunnan, China
Angelica Sinensis	AS08	National Institutes for Food and Drug Control	"digital identity"; mixed sample	Gansu, China
Angelica Sinensis	AS09	National Institutes for Food and Drug Control	"digital identity"; mixed sample	Qinghai, China
Angelica Sinensis	AS10	National Institutes for Food and Drug Control	Identification verification; mixed sample	Ningxia, China
Levisticum Officinale	LO01	National Institutes for Food and Drug Control	"digital identity"; mixed sample	Henan, China
Levisticum Officinale	LO02	National Institutes for Food and Drug Control	"digital identity"; mixed sample	Hebei, China
Levisticum Officinale	LO03	National Institutes for Food and Drug Control	"digital identity"; mixed sample	Liaoning, China
Levisticum Officinale	LO04	National Institutes for Food and Drug Control	"digital identity"; mixed sample	Shanxi, China
Levisticum Officinale	LO05	National Institutes for Food and Drug Control	"digital identity"; mixed sample	Shan'Xi, China
Levisticum Officinale	LO06	National Institutes for Food and Drug Control	"digital identity"; mixed sample	Shandong, China
Levisticum Officinale	LO07	National Institutes for Food and Drug Control	"digital identity"; mixed sample	Shanxi, China
Levisticum Officinale	LO08	National Institutes for Food and Drug Control	"digital identity"; mixed sample	Shanxi, China
Levisticum Officinale	LO09	National Institutes for Food and Drug Control	"digital identity"; mixed sample	Neimenggu, China
Levisticum Officinale	LO10	National Institutes for Food and Drug Control	Identification verification; mixed sample	Jiangsu, China
Angelica Gigas	AG01	National Institutes for Food and Drug Control	"digital identity"; mixed sample	Yuanshan, Korea
Angelica Gigas	AG02	National Institutes for Food and Drug Control	"digital identity"; mixed sample	Jilin, China
Angelica Gigas	AG03	National Institutes for Food and Drug Control	"digital identity"; mixed sample	Liaoning, China
Angelica Gigas	AG04	National Institutes for Food and Drug Control	"digital identity"; mixed sample	Heilongjiang, China
Angelica Gigas	AG05	National Institutes for Food and Drug Control	"digital identity"; mixed sample	Neimenggu, China
Angelica Gigas	AG06	National Institutes for Food and Drug Control	Identification verification; mixed sample	Jilin, China
Angelica Acutiloba	AA01	National Institutes for Food and Drug Control	"digital identity"; mixed sample	Jilin, China

Angelica Acutiloba	AA02	National Institutes for Food and Drug Control	"digital identity"; mixed sample	Hubei, China
Angelica Acutiloba	AA03	National Institutes for Food and Drug Control	"digital identity"; mixed sample	Hokkaido, Japan
Angelica Acutiloba	AA04	National Institutes for Food and Drug Control	"digital identity"; mixed sample	Hokkaido, Japan
Angelica Acutiloba	AA05	National Institutes for Food and Drug Control	"digital identity"; mixed sample	Jilin, China
Angelica Acutiloba	AA06	National Institutes for Food and Drug Control	Identification verification; mixed sample	Yuanshan, Korea
0 % LO	MixLO01	self-preparation	Identification verification	—
3 % LO	MixLO02	self-preparation	Identification verification	—
5 % LO	MixLO03	self-preparation	Identification verification	—
10 % LO	MixLO04	self-preparation	Identification verification	—
20 % LO	MixLO05	self-preparation	Identification verification	—
50 % LO	MixLO06	self-preparation	Identification verification	—
100 % LO	MixLO07	self-preparation	Identification verification	—
0 % AG	MixAG01	self-preparation	Identification verification	—
3 % AG	MixAG02	self-preparation	Identification verification	—
5 % AG	MixAG03	self-preparation	Identification verification	—
10 %AG	MixAG04	self-preparation	Identification verification	—
20 % AG	MixAG05	self-preparation	Identification verification	—
50 % AG	MixAG06	self-preparation	Identification verification	—
100 % AG	MixAG07	self-preparation	Identification verification	—
0 % AA	MixAA01	self-preparation	Identification verification	—
3 % AA	MixAA02	self-preparation	Identification verification	—
5 % AA	MixAA03	self-preparation	Identification verification	—
10 %AA	MixAA04	self-preparation	Identification verification	—
20 % AA	MixAA05	self-preparation	Identification verification	—
50 % AA	MixAA06	self-preparation	Identification verification	—
100 % AA	MixAA07	self-preparation	Identification verification	—
Blind market AS materials	BMS01	Company Code: BZYG	Identification analysis	—
Blind market AS materials	BMS02	Company Code: HH	Identification analysis	—
Blind market AS materials	BMS03	Company Code: HM	Identification analysis	—

Blind market AS materials	BMS04	Company Code: KL	Identification analysis	—
Blind market AS materials	BMS05	Company Code: MS	Identification analysis	—
Blind market AS materials	BMS06	Company Code: QCT	Identification analysis	—
Blind market AS materials	BMS07	Company Code: HS	Identification analysis	—
Blind market AS materials	BMS08	Company Code: SM	Identification analysis	—
Blind market AS materials	BMS09	Company Code: XT	Identification analysis	—

Table S2 The ion information of "matrix characteristics" of Angelica gigas

Rt	*m/z*	I	*Rt*	*m/z*	I	*Rt*	*m/z*	I
18.15	329.1397	2822643	18.01	329.1403	415743	20.63	1055.3843	209085
14.84	331.1536	1852610	23.20	612.4487	402734	24.00	761.5473	207886
6.48	247.0972	1843738	14.48	185.0606	387548	5.72	187.0399	206555
20.80	671.3800	1480215	21.88	875.5790	374622	14.64	227.0722	201913
23.00	873.5711	1334248	22.95	725.4755	374081	14.60	329.4431	200105
14.61	247.0975	1295410	20.63	1034.4546	371549	6.91	495.1509	194014
14.49	247.0975	1286159	16.96	721.3231	367383	6.91	247.0976	193298
14.53	329.3367	1074712	14.69	330.5948	350825	14.45	684.2313	192379
17.68	689.2985	1057161	20.59	657.2710	347473	14.62	229.1670	192251
23.03	903.5789	963094	20.21	1071.4347	343387	23.10	970.5656	192011
6.49	989.2948	904014	14.64	676.2447	331453	18.10	657.2716	184451
14.59	329.3398	885949	13.40	315.1247	330293	20.10	1034.4543	183165
23.04	873.6008	864126	22.15	937.6129	305977	15.73	343.1552	176218
18.17	727.2525	855519	5.72	247.2560	295388	23.42	717.4949	175235
18.18	711.2788	823539	14.51	676.2448	291148	17.09	721.3233	173597
18.27	706.3232	796788	22.31	673.4071	290197	5.75	562.2416	172821
13.73	317.1391	791686	20.09	1087.4114	270995	18.16	789.3616	172118
18.27	706.3243	789360	18.00	711.2789	268031	14.30	571.3788	171497
17.86	689.2976	682270	21.54	1033.6652	261194	22.28	685.4440	171385
6.21	409.1508	661149	11.78	345.1343	254532	20.06	1049.4608	168953
18.27	711.2783	597838	24.21	854.6054	249032	25.66	772.5545	167742
14.47	229.2472	584586	20.63	1039.4109	240423	17.09	299.1647	162634
16.83	721.3233	520774	18.15	734.3540	235963	8.92	363.1441	161138
14.58	684.2317	492162	12.30	231.2643	233974	14.45	668.2558	160389
18.00	706.3250	489849	24.35	854.6090	225693	24.02	761.5095	155392
11.88	345.1344	468831	14.61	230.3652	222548	15.76	343.1558	155369
18.28	727.2526	467243	14.47	229.1671	219211	20.82	716.4684	155302
13.57	315.1248	462374	14.49	230.3653	214308	22.65	483.3645	155239
12.82	345.1358	458539	10.29	345.1350	214024	14.95	343.1184	150745
14.62	229.2478	455793	14.84	331.3439	213036	21.58	932.6952	150672
20.09	1066.4824	437808	21.61	932.6592	212833	14.61	201.0920	149993
18.02	727.2551	430146	18.57	657.2706	212454	14.49	231.4766	147976
6.50	229.0868	420246	17.96	657.2716	211126	5.72	247.3614	147486
22.75	699.4567	146826	—	—	—	—	—	—

Table S3 The ion information of "matrix characteristics" of Angelica Acutiloba

Rt	*m/z*	I	*Rt*	*m/z*	I	*Rt*	*m/z*	I
23.02	1013.6939	1084650	22.67	746.5610	61240	25.73	680.6153	32938
22.98	729.5584	476953	24.08	516.4297	60060	22.18	547.3797	32706
21.53	760.5833	465991	24.97	213.1136	59989	23.55	555.4236	31891
22.97	900.6759	446470	16.86	383.2794	59663	23.12	542.4203	31877
25.01	1011.7160	399439	22.94	861.4743	58960	7.90	626.4252	31235
24.92	1011.6958	298289	20.50	547.4615	57364	25.93	680.6178	31025
24.57	687.4640	218212	25.60	869.6199	55850	6.50	481.0973	30443
24.84	165.0970	209917	21.36	498.4307	54895	19.71	754.4621	30368

24.12	165.1054	208756	23.17	306.2792	53703	22.64	517.3349	30296
24.04	1009.7514	201302	24.76	725.4958	51952	20.96	641.4836	29757
25.96	613.4807	201105	24.67	725.5749	50710	25.94	1027.7452	29635
24.04	1009.7098	188341	23.02	498.3824	48008	24.91	725.5691	29102
24.77	796.5522	180342	17.67	585.3578	45244	21.98	714.4024	29077
24.24	847.6147	168313	10.50	207.0659	44073	20.46	494.3976	28519
24.19	1009.7049	149561	17.33	642.3791	43518	9.36	220.3014	28249
23.00	503.3434	142429	23.75	617.4091	42404	19.84	562.4584	28122
25.61	1009.7134	130466	14.69	609.4101	41998	21.53	522.3780	27860
3.22	163.0395	125332	24.19	165.0932	40983	2.17	194.0837	27815
23.05	699.5374	122876	25.69	937.6907	39886	25.91	1027.7779	27433
25.71	869.6237	102816	24.90	741.4800	39354	18.05	456.3148	26468
21.35	500.4456	99709	5.36	510.1977	38831	21.68	872.6249	26178
23.02	699.5063	94958	14.54	301.1076	37422	5.72	427.1540	25956
2.22	542.1989	94622	18.61	705.4840	36845	18.57	634.4165	25339
17.67	431.3531	94450	21.50	1070.7305	35903	21.98	686.3723	25315
25.80	613.4814	89923	7.96	304.1188	35588	23.84	500.4442	24264
23.15	606.4382	80206	20.41	415.2636	34704	18.56	639.3720	24247
22.63	746.5584	79764	25.75	1026.7662	34641	24.02	673.5764	24065
25.61	881.6019	79689	25.17	1011.6792	33988	5.58	540.2099	23846
22.97	511.3258	79241	23.16	857.5469	33956	5.44	702.2759	23824
21.55	901.5934	73930	22.20	778.4373	33796	23.59	469.3334	23474
24.08	901.6685	68214	20.85	483.2939	33754	4.61	702.2450	23280
23.32	517.3337	67974	15.99	627.3362	33673	23.17	712.5372	23132
23.52	709.5847	65290	23.86	631.1137	33053	24.35	749.4263	23125
5.92	177.0555	62750	—	—	—	—	—	—

Table S4 The ion information of "matrix characteristics" of Levisticum Officinale

Rt	*m/z*	I	*Rt*	*m/z*	I	*Rt*	*m/z*	I
17.67	761.4066	1962137	23.24	695.3787	183956	23.00	658.4109	119428
24.59	869.4446	681381	22.20	649.3538	182195	21.47	686.4412	118426
24.56	876.5408	673389	24.96	691.4438	181659	24.28	700.4552	118106
21.28	191.1085	563857	23.08	675.4106	177085	21.79	785.4033	117510
23.16	876.5138	549874	21.64	845.4048	169178	24.05	637.3883	117266
22.05	191.1073	516164	18.69	205.1240	169154	18.54	417.2054	112711
24.61	848.5116	467097	23.26	595.3584	168402	21.60	630.3800	111592
16.68	191.1082	453984	21.63	686.4410	168263	22.64	728.4673	110820
23.45	605.3634	442964	24.65	811.5138	166725	19.72	691.4196	110699
25.02	623.3744	424093	21.46	658.4102	166656	25.00	733.4439	110554
19.57	191.1079	401573	20.76	669.3790	165819	25.75	931.5480	108384
20.59	191.1081	378417	25.71	853.4665	165763	23.70	642.4059	107779
19.92	631.4072	364293	21.32	641.3694	164860	20.26	395.2231	106321
25.65	954.5684	358593	19.28	191.1080	162766	24.49	745.4335	104542
22.43	191.1071	349480	22.26	191.1072	160996	24.99	733.4457	104501
17.66	382.2110	340576	24.03	770.4949	160749	23.15	534.3523	103088
25.07	970.7680	323662	24.78	995.6236	160142	22.22	686.4387	102614
25.09	915.5560	321717	21.81	700.4329	158966	20.51	697.3657	102577
24.93	970.7684	317366	23.97	776.4723	157728	16.10	343.2365	100431
17.76	382.2113	315016	18.69	427.2495	154475	21.70	801.3802	100025

25.15	915.5496	297234	24.15	637.3884	151168	23.85	642.4018	99989
22.67	672.4174	273093	25.04	686.4430	145451	21.46	672.4016	99937
21.68	785.3955	266863	23.59	675.3724	140187	19.11	634.3388	99617
21.61	585.3220	237434	21.63	700.4358	137771	25.00	682.4513	99534
21.98	738.4449	218945	23.42	700.4531	133688	16.10	152.0631	99139
24.62	931.5467	214499	16.83	191.1078	132899	22.71	618.3554	98539
20.61	865.5435	205265	19.47	393.2239	132390	18.80	417.2104	97709
18.84	205.1239	203435	16.09	713.4600	131457	23.15	561.3687	96989
21.80	630.3781	202908	23.17	786.4661	130619	20.50	928.5234	95895
20.64	865.5436	198358	23.17	848.5058	129325	25.61	733.4478	95232
22.08	635.3450	189824	21.32	595.3399	127016	23.29	876.5350	95075
23.13	653.4119	188691	20.21	809.4796	125558	25.69	825.5241	93436
22.77	848.5046	188177	23.58	942.7296	122951	23.02	986.7607	92599
21.83	191.1074	184100	—	—	—	—	—	—

Table S5 The ion information of "matrix characteristics" of Angelica Sinensis

Rt	*m/z*	I	*Rt*	*m/z*	I	*Rt*	*m/z*	I
18.71	570.5090	772364	17.18	426.2642	59634	7.35	332.0916	34096
23.45	756.5078	314366	23.82	901.5502	59183	19.99	671.3687	33814
17.52	761.4059	225974	23.28	623.3735	53569	17.71	427.2826	33593
23.32	756.5123	221275	24.76	597.3857	52726	23.62	737.4409	32903
21.75	593.2873	215737	21.60	843.4818	52461	23.96	656.3957	32535
25.09	687.4493	193947	23.15	921.5704	50943	20.53	655.3602	32256
25.74	671.4848	182232	23.42	777.4668	48960	21.85	890.4029	31477
17.17	783.3878	172083	23.69	637.3611	48782	23.05	731.4430	31444
15.45	189.0918	164037	25.24	828.6089	48625	22.54	594.3756	30348
23.33	663.3654	154489	17.17	799.3619	48047	23.39	608.3931	30321
24.92	931.5492	145965	15.46	311.1263	46430	5.63	353.1582	30253
24.27	921.5789	136333	21.70	843.5179	46091	25.64	597.4294	30209
17.33	761.4071	129979	21.68	927.5743	45941	16.55	341.2103	30056
22.80	927.5941	120654	22.75	738.4298	45575	24.24	699.4401	29792
24.53	701.4580	120180	23.27	925.6642	44262	22.66	477.3350	29701
23.14	691.4348	119002	23.92	979.6189	44212	23.72	511.3417	29583
21.76	989.5844	118444	21.55	927.5520	43294	23.07	821.4276	29243
24.85	853.4685	113099	22.03	655.3671	40611	23.05	925.6620	28605
23.30	705.4704	106523	24.86	869.4552	39958	21.85	895.3497	27501
21.67	723.4353	95935	22.58	1135.4563	39741	4.39	342.1693	27087
22.76	659.4003	94368	22.42	607.4024	39127	20.09	327.2332	26929
20.73	480.4388	91663	21.55	643.4048	39098	15.01	325.2172	26910
25.42	843.5653	86820	17.20	145.1014	38504	23.01	571.3035	26633
25.02	931.5494	85598	5.81	587.1738	38045	19.69	754.3685	26409
23.16	759.4610	80028	23.00	925.6065	37143	19.61	833.4291	26350
23.18	548.4254	78666	18.25	279.2327	37142	22.75	580.3770	26074
24.72	853.4711	78340	23.33	738.4251	37012	20.68	663.4041	25834
23.24	585.3820	74430	21.58	918.4938	36998	24.64	679.3426	25781
23.25	747.4108	73891	22.64	656.3961	36258	18.52	501.2258	25627
22.15	671.3775	72125	20.20	655.3610	35757	21.66	327.2320	25505
23.21	740.5198	64397	15.12	325.2174	35185	23.22	731.4609	25503
23.20	499.3176	62674	20.80	731.4265	35179	25.01	703.4353	25031

22.79	843.4812	60952	23.92	673.4445	34405	24.29	657.4517	24895
25.30	843.5623	59822	—	—	—	—	—	—

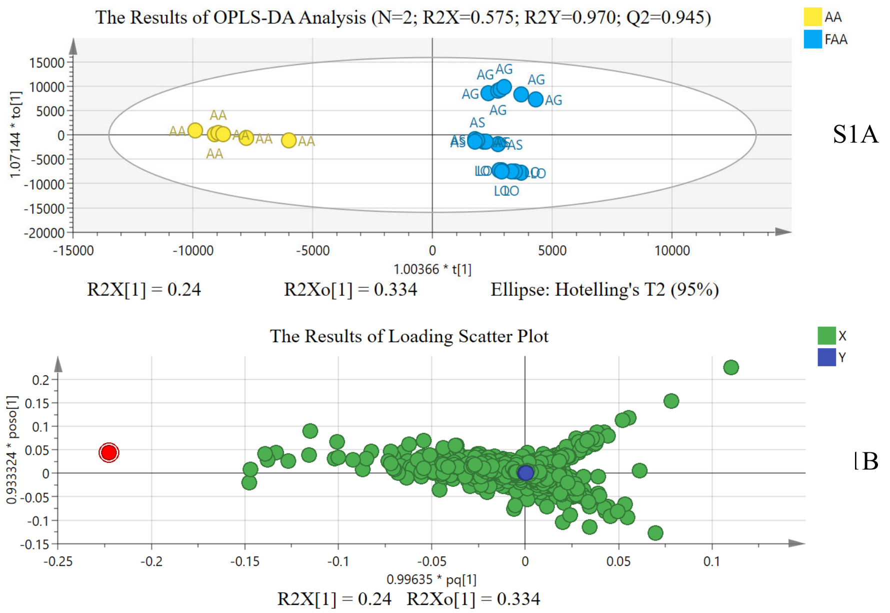

Fig.S1 The results of chemometric analysis of AA and non-AA (A): The results of OPLS-DA analysis; (B): The results of loading scatter plot)

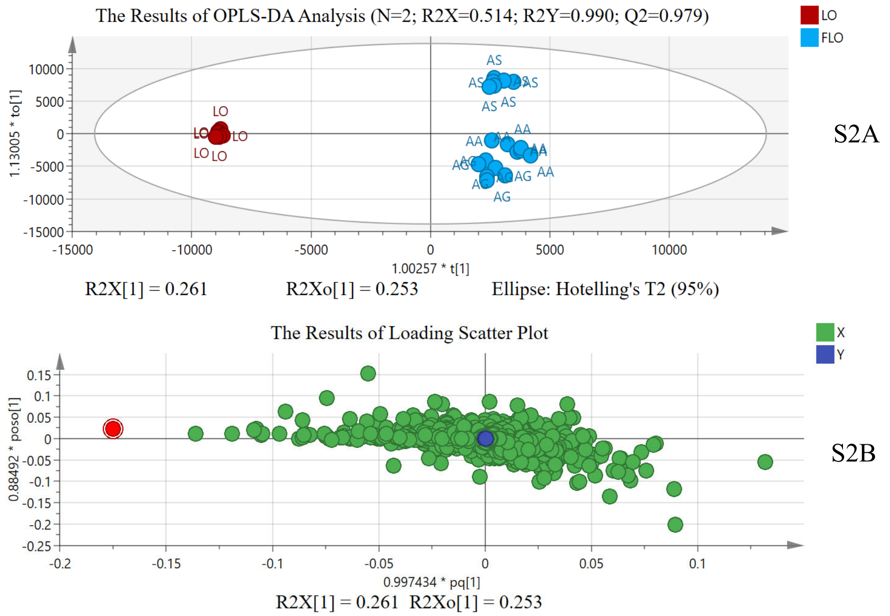

Fig.S2 The results of chemometric analysis of LO and non-LO (A): The results of OPLS-DA analysis; (B): The results of loading scatter plot)

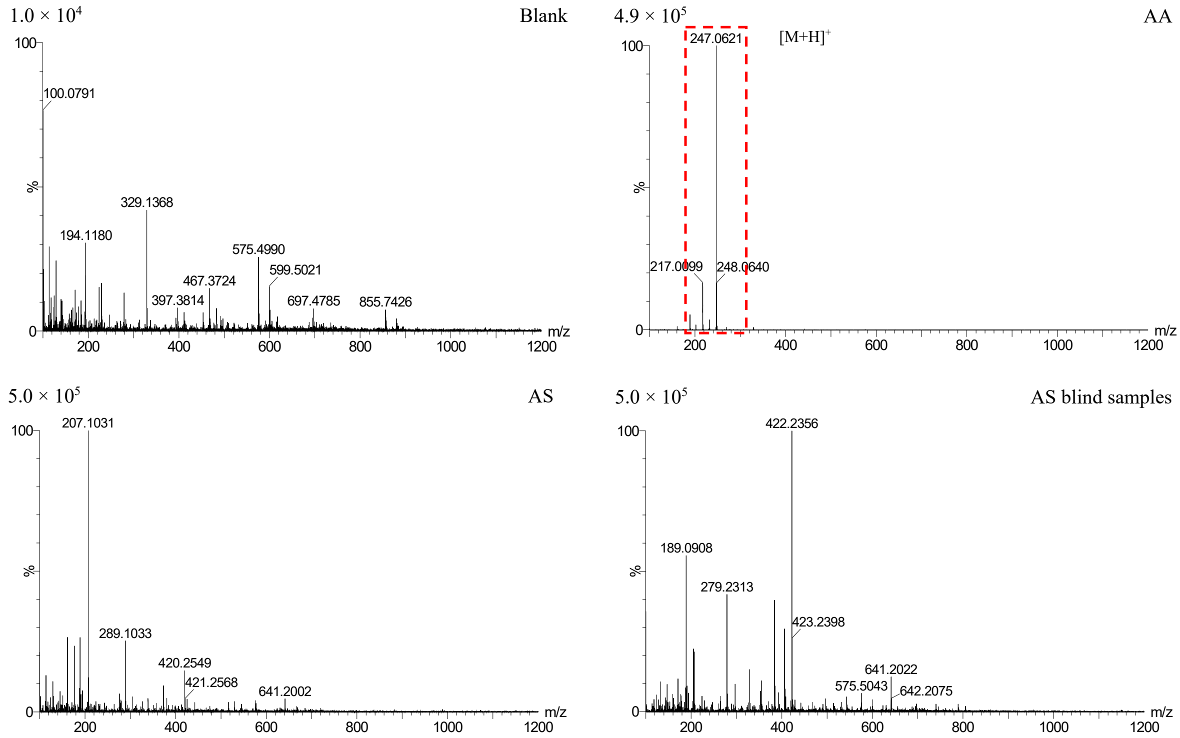

Fig.S3 The extraction results of 10.31 min_247.0621 *m/z* in different samples

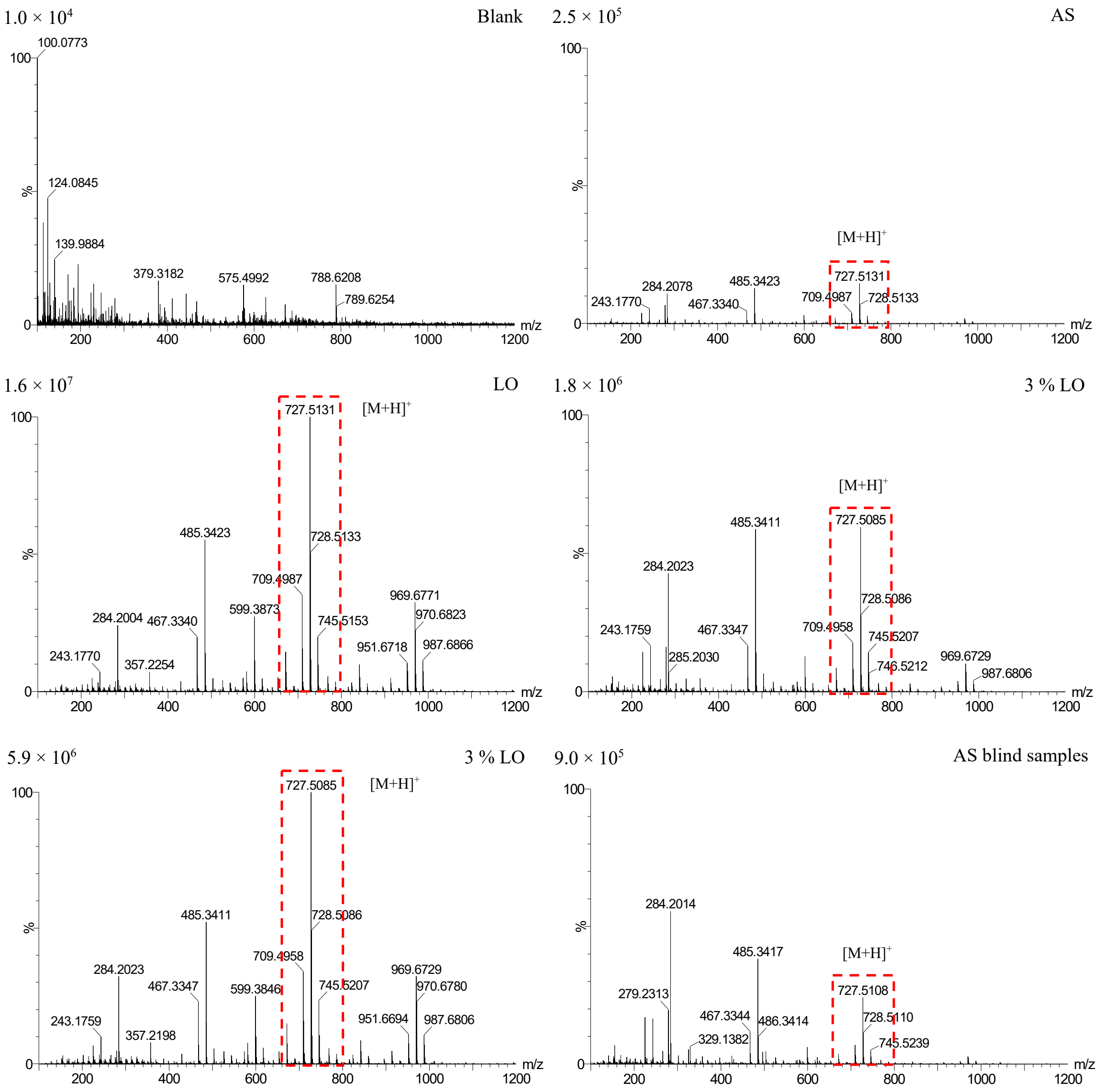

Fig.S4 The extraction results of 16.10 min_727.5131 *m/z* in different samples
